# Compatibility of preparatory procedures for the analysis of cortisol concentrations and stable isotope (*δ*^13^C, *δ*^15^N) ratios: a test on brown bear hair

**DOI:** 10.1093/conphys/cox021

**Published:** 2017-03-24

**Authors:** Agnieszka Sergiel, Keith A. Hobson, David M. Janz, Marc Cattet, Nuria Selva, Luciene Kapronczai, Chantel Gryba, Andreas Zedrosser

**Affiliations:** 1 Institute of Nature Conservation, Polish Academy of Sciences, 31120 Krakow, Poland; 2 Environment Canada, 11 Innovation Blvd., Saskatoon, Saskatchewan, CanadaS7N 3H5; 3 Department of Biology, University of Western Ontario, London, Ontario, CanadaN6A 5B7; 4 Department of Veterinary Biomedical Sciences, University of Saskatchewan, Saskatoon, Saskatchewan, CanadaS7N 5B4; 5 RGL Recovery Wildlife Health & Veterinary Services, Saskatoon, Saskatchewan, CanadaS7H 4A6; 6 Department of Veterinary Pathology, University of Saskatchewan, Saskatoon, Saskatchewan, CanadaS7N 5B4; 7 Toxicology Centre, University of Saskatchewan, Saskatoon, Saskatchewan, CanadaS7N 5B3; 8 Department of Natural Sciences and Environmental Health, University College of Southeast Norway, 3800 Bø, Norway; 9 Institute for Wildlife Biology and Game Management, University for Natural Resources and Life Sciences, 1180 Vienna, Austria

**Keywords:** Dietary intake, laboratory procedures, stable isotopes, stress, *Ursus arctos*, wildlife physiological ecology

## Abstract

The measurement of naturally occurring glucocorticoids and stable isotopes of several elements has gained importance in wildlife studies in recent decades and opened a myriad of ecological applications. Cortisol and stable isotopes equilibrate in animal tissues over periods of integration related to the growth rate of the tissue, providing information reflecting systemic cortisol secretion and dietary intake. Sample preparation shares the common step of first cleaning the sample of external contamination. However, it is not well understood how different solvents used in sample preparation affect isotopic and cortisol values, and whether it is safe to follow the same procedures for both measures to optimize analyses of the same sample. We conducted an experiment to compare different preparation protocols for the analysis of cortisol concentrations and stable carbon (*δ*^13^C) and nitrogen (*δ*^15^N) isotope ratios in hair. Hair samples from 12 brown bears (*Ursus arctos*) were each divided into five aliquots; two aliquots were rinsed with a 2:1 chloroform:methanol (v/v) mixture with one aliquot ground prior to cortisol analysis and the other left intact for stable isotope analyses; two aliquots were washed with methanol with one aliquot ground prior to cortisol analysis and the other left intact for stable isotope analyses; and one aliquot washed with methanol and ground prior to stable isotope analyses. The cortisol, *δ*^13^C and *δ*^15^N values remained consistent following all treatments. Our results indicate that hair samples rinsed with a 2:1 chloroform:methanol mixture or washed with methanol can be used for both types of analyses. Further, hair that has been ground in a standard hair cortisol procedure can also be used for stable isotope analysis. This information is important for improving laboratory efficiency and compatibility of procedures used for wildlife physiological ecology studies where concurrent measurements of cortisol and stable isotopes in hair are required.

## Teaser

Stress hormone levels and stable isotope ratios in hair or feathers represent an integrated picture of hormone physiology and foraging ecology of an animal. We evaluate the compatibility of laboratory preparation procedures used in ecophysiology with the intention of optimizing the efficiency of future research.

## Introduction

Levels of stress hormones and stable isotope ratios in animal tissues are increasingly recognized as important tools in studies on the physiology and ecology of wildlife (e.g. Kempster *et al*., 2007; Barger and Kitaysky, 2011; Deschner *et al*., 2012; Bryan *et al*., 2013). Keratinous tissues, such as hair and feathers, offer the longest record of an animal's circulating glucocorticoid (GC) concentrations (Bortolotti *et al*., 2009; Macbeth *et al*., 2010; Jenni-Eiermann *et al*., 2015). Steroids are incorporated into the hair shaft and feather during their growth, and therefore, GC levels in those matrices are thought to reflect average systemic levels over the respective growth phase by integrating baseline levels and elevated adrenocortical secretion (Cattet *et al*., 2014; Jenni-Eiermann *et al*., 2015). Hair or feather GC concentrations are increasingly applied to evaluate chronic exposure to various stressors or potentially stressful conditions (Koren *et al*., 2002; Davenport *et al*., 2006; Accorsi *et al*., 2008; Macbeth *et al*., 2010; Comin *et al*., 2011; Fairhurst *et al*., 2014). Additionally, GCs in those sample matrices are stable over time and resistant to environmental exposure (Bortolotti *et al*., 2009; Macbeth *et al*., 2010). Therefore, measuring GC concentration provides a useful tool in understanding the role of stress in the life history, conservation physiology, health, and ecology of wildlife species (e.g. Wikelski and Cooke, 2006; Macbeth *et al*., 2010; Cattet *et al*., 2014).

Stable isotopes are also incorporated over time into hair and feathers (Wassenaar, 2008; Bontempo *et al*., 2014). Stable nitrogen (*δ*^15^N) and carbon (*δ*^13^C) isotope ratios are widely employed to reconstruct and assess temporal and spatial variation in diet using extant or archived materials (Newsome *et al*., 2007; Crawford *et al*., 2008). These isotopes are also used to characterize trophic niche and community structure (Boecklen *et al*., 2011), elucidate animal migration and movements (Hobson and Wassenaar, 2008), and determine individual specialization and habitat selection (Newsome *et al*., 2009). Stable isotope analyses are also employed in wildlife forensics and ecotoxicological studies (Bowen *et al*., 2005). Combining concurrent measures of long-term stress and stable isotopes within the same study may open up a wide selection of testable ecological theories, including relationships between diet and stress (e.g. Bryan *et al*., 2013; Fairhurst *et al*., 2014; Lafferty *et al*., 2015).

Compared to blood, saliva, urine or feces, hair and feathers are media that can be transported and stored at room temperature. The use of hair and feathers for quantifying stress offers other desirable features, such as non-invasive collection and retrospective analyses of stress using archived samples (Cattet *et al*., 2014). Additionally, some studies have made use of hair collected from museum and archeological specimens to determine cortisol levels (Webb *et al*., 2010; Bechshøft *et al*., 2012). However, the preparatory methods for the measurement of cortisol concentration differ from the methods used for the determination of stable isotope values, although both sets of measurements may be desired within the same study.

For cortisol analysis, hair is commonly washed and extracted using methanol, and ground into a fine powder prior to extraction (Macbeth *et al*., 2010). For stable isotope analysis (SIA), hair samples are typically washed in a 2:1 chloroform:methanol (v/v) mixture (e.g. Riofrío-Lazo and Páez-Rosas, 2015), and then cut into small pieces (Newsome *et al*., 2010) or powdered (Darimont *et al*., 2007; Riofrío-Lazo and Páez-Rosas, 2015). Because cortisol concentrations in blood and sweat are often far greater than those found in hair, failure to adequately wash the external surface of hair shafts contaminated with these substances will falsely elevate measurement of the internal hair shaft (medullar) cortisol concentration (e.g. Stalder and Kirschbaum, 2012). Similarly, the single wash that is often used for stable isotope analyses may not be sufficient to remove external contamination of the hair shaft with blood and/or perspiration (Russel *et al*., 2014). On the other hand, if a solvent is too aggressive or if hair is immersed in a solvent for prolonged time, hair shaft cortisol may be lost if the solvent fully penetrates the hair cuticle (Eser *et al*., 1997). Stable isotope values may also be affected by sample processing procedures (Font *et al*., 2007). Carbon composition of surface oils and waxes can be significantly different from pure tissue composition and thus may influence stable isotope values. Therefore, in cleaning protocols for fixed tissue samples, volatile solvent mixtures are used to remove surface lipids (Wassenaar, 2008). An important question that remains is whether these preparatory procedures used for washing and/or grinding hair can be merged into a single protocol to increase the efficiency of sample preparations, and to reduce labor input and costs for research projects.

It is unknown whether the preparation process for *δ*^13^C and *δ*^15^N analysis affects the reliable measurement of cortisol concentration in hair and *vice versa*. In this study, we compared commonly used, but different, wash and sample processing procedures to determine their effect on cortisol and stable isotope values measured in hair. We used hair samples collected from brown bears (*Ursus arctos*) as a model system. Here, we evaluate whether different preparatory procedures yielded consistent results. Specifically, we test the null hypotheses that (1) there are no differences in cortisol concentrations or (2) stable isotope ratios (*δ*^13^C and *δ*^15^N) between brown bear hair samples prepared using standard washing procedures in cortisol (methanol wash) or stable isotope ratio (2:1 chloroform:methanol rinse) measurement protocols, and that (3) there are no differences in stable isotope ratios between hair samples washed following the procedure in cortisol concentration measurement protocol and left intact or ground into powder before stable carbon and nitrogen ratio measurement.

## Material and methods

### Sample selection

We selected guard hair samples from 12 brown bear individuals from a larger pool of archived samples available, based on the criteria that the samples (1) were of sufficient quantity for further subdivision, (2) originated from the same body region, and (3) were free of visible external debris (e.g. blood, dirt). Samples were collected by plucking from the top of the shoulders (the hump) of bears captured in Sweden (1993–1996), or by cutting close to skin at the top of the neck for bears captured in Poland (2014–2015), and were stored dry in paper envelopes in the dark at room temperature. All samples were collected for ongoing long-term monitoring projects. Bear capture, immobilization and handling were carried out as described in Arnemo *et al*. (2012), and approved by the appropriate authorities (Sweden: Swedish Environmental Protection Agency, Stockholm, #NV-0758-14, and the Swedish Board of Agriculture, #31-11 102/12; Poland: General Directorate for Environmental Protection, Warsaw, #DOP-OZ.6401.08.2.2013.ls.1) and Ethical Committees (Sweden: Swedish Ethical Committee on Animal Research, Uppsala, #C18/15; Poland: I Local Ethical Committee in Krakow, #21/2013).

### Sample preparation and procedures

#### Preparation of hair samples

**Figure 1:**
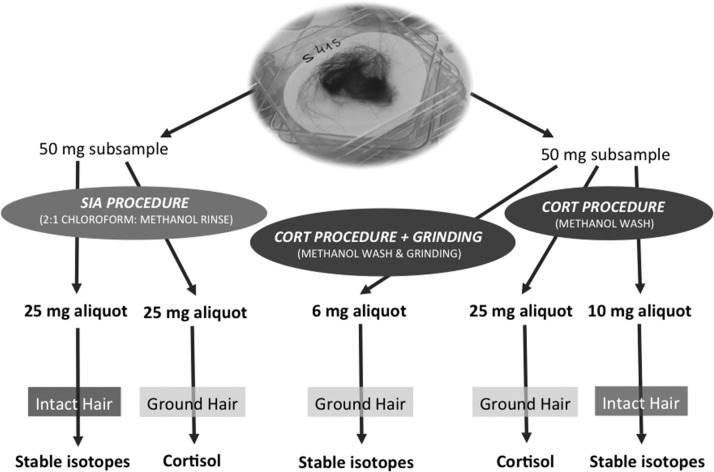
The breakdown of a single hair sample (≥100 mg) collected from an individual bear into 50 mg subsamples, followed by smaller aliquots, and then processed accordingly following preparation procedures for cortisol (CORT) and stable isotope analyses (SIA; as indicated by arrows).

We selected two subsamples (≥50 mg per subsample) that appeared similar with respect to length, color, and amount of external contamination from each of 12 samples of guard hair. The hair follicles were excised from all plucked hair samples to ensure that cortisol and stable isotope measurements were based on hair shafts only. Each subsample was further subdivided into two aliquots; one to be used for cortisol analysis and the other for SIA. Aliquots were washed with methanol (CORT procedure) or rinsed with 2:1 chloroform:methanol (SIA procedure) and processed accordingly for cortisol and stable isotope analyses as shown in Fig. 1. Hair aliquots for cortisol measurement were ground prior to analysis using a Retsch MM 301 Mixer Mill (Retsch Inc., Newtown, Pennsylvania, USA), whereas hair for stable isotope analyses were left as intact hair shafts, with the exception of one 6-mg aliquot that was ground and set aside. This aliquot was used to test the effect of the full CORT procedure (both methanol wash and grinding) on stable isotope ratios.

#### CORT preparatory procedure

The CORT preparatory procedure is a standard protocol used for hair cortisol analysis that involves washing hair samples with HPLC-grade methanol in three cycles of 3 min each (Macbeth *et al*., 2010). The washed hair is then air-dried thoroughly for 24 h at room temperature. Three aliquots per bear were prepared following this protocol (Fig. 1). A 10-mg aliquot was left intact and used for SIA, and the rest was ground into powder, according to the protocol for cortisol analysis. From the powdered hair, a 25-mg aliquot was used for cortisol analysis, and a 6-mg aliquot for SIA (Fig. 1).

#### SIA preparatory procedure

The SIA preparatory procedure is a standard protocol for SIA that involves rinsing hair samples with a 2:1 (v/v) mixture of chloroform:methanol (Hobson *et al*., 2000). The hair is then removed from the solvent and air-dried thoroughly for 24 h at room temperature. Two 25 mg aliquots per bear were prepared following this protocol with one aliquot left intact and used for SIA and the other was ground and used for cortisol analysis (Fig. 1).

### Hair cortisol analysis

For cortisol analysis, we used 24 aliquots of hair (2 × 25 mg aliquots per bear) that were washed using either the SIA (chloroform:methanol rinse; one per bear) or CORT (methanol wash; one per bear) procedure. Aliquots of 25 mg were combined with 0.5 ml of HPLC grade methanol for 24 h on a slow rotator to extract cortisol. Subsequent procedures and cortisol analysis were conducted in accordance with the protocol described by Macbeth *et al*. (2010). The extract was centrifuged for 15 min at 2150*g*, the methanol was removed, evaporated until dryness under nitrogen gas (38°C), and reconstituted in phosphate buffer (0.2 ml). Cortisol was quantified using a commercially available enzyme immunoassay (EIA) kit previously validated for use with brown bear hair (Oxford Biomedical, Lansing, Michigan, USA; Macbeth *et al*., 2010). Extracts were run in duplicates with intra-assay coefficient of variability (CV) of 4.6%, calculated from the individual CVs for all of the duplicates.

### Stable isotope analysis

For SIA, we used 24 aliquots of intact hair that were washed using either the SIA (10 mg per bear) or CORT (25 mg per bear) procedures, and additionally 12 aliquots (6 mg per bear) prepared according to the CORT procedure and ground into powder. Subsequent *δ*^13^C and *δ*^15^N analyses were conducted in accordance with the protocol described in Hobson *et al*. (2000). Portions (1 mg) of each aliquot were weighed into tin capsules and combusted in a Carlo Erba elemental analyzer (Milan, Italy) interfaced with a Europa 20:20 continuous flow mass spectrometer. Results are reported in delta (*δ*) notation as parts per thousand (‰) deviation from international standards, Vienna Pee dee Belemnite (VPDB) for *δ*^13^C and atmospheric (AIR) for *δ*^15^N. Based on within-run (*n* = 12) replicate measurements of an egg albumen standard, we estimated measurement error to be ±0.1‰ and ±0.2‰ for *δ*^13^C and *δ*^15^N, respectively.

### Statistical analysis

Data distribution was checked using the Shapiro–Wilk test and the assumption of normality was not fulfilled. Therefore, a non-parametric pairwise test (Wilcoxon signed-rank test) was used to compare the cortisol concentration in hair from the same individual (*n* = 12; Supplementary table 1) prepared by either CORT or SIA washing protocols (Fig. 1). We used the same non-parametric test to compare *δ*^13^C and *δ*^15^N values obtained by preparing hair samples of the same individual (*n* = 12; Supplementary table 1) by the SIA washing and the CORT washing procedure, as well as the CORT washing procedure followed by grinding of the hair sample (referred to as CORT/grind in the results). We did not apply sequential Bonferroni corrections in the pairwise tests, because this method is unnecessarily conservative (e.g. Narum, 2006). In addition, all of our test results had *P*-values far above 0.05 (see Results), which we considered as statistically significant. We present results for all analyses as median (range) as well as mean ± SD. All tests were carried out in the statistical software R 3.3.2 (R Core Team, 2016).

A comparison of *δ*^13^C, *δ*^15^N (‰) and cortisol concentrations (pg/mg) values measured in hair samples of all individuals, descriptive statistics, concordance correlation coefficients, as well as further analyses comparing the effect of cleaning procedures on carbon and nitrogen fraction (%) and carbon:nitrogen ratio are included as Supplementary material.

## Results

**Figure 2:**
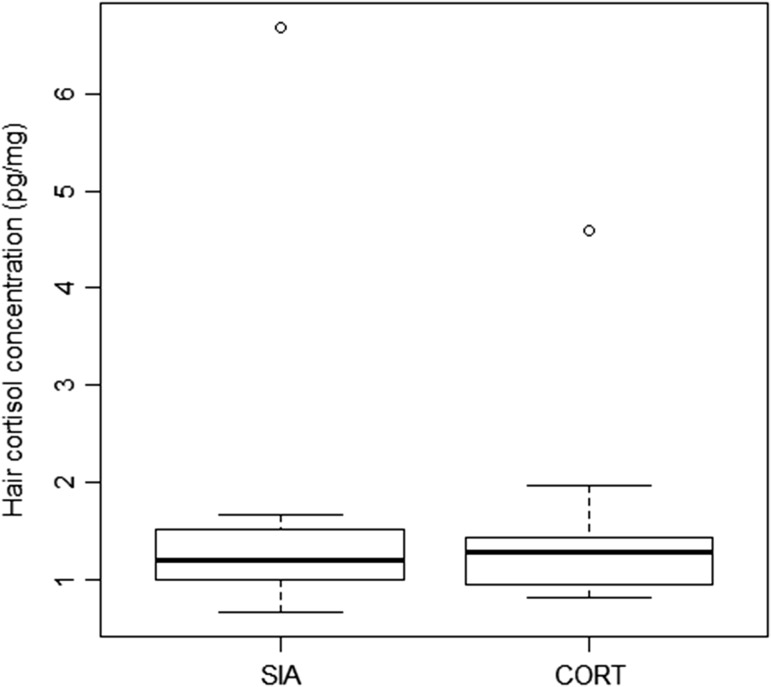
Box plot of median cortisol level (in picograms per milligram) in hair samples of 12 brown bear individuals across different washing protocols used in preparatory stage. The cortisol concentrations in aliquots rinsed with 2:1 chloroform:methanol mixture, as typical in SIA, were not significantly different from aliquots washed in methanol, as typically used in cortisol concentration analysis (CORT), by the Wilcoxon test (*V* = 36, *P* = 0.884).

We found no significant difference (*V* = 36, *P* = 0.884) between the pairwise cortisol concentrations of hair washed using the SIA procedure (1.19 (0.66, 6.69); 1.62 ± 1.63 pg/mg) compared to the CORT procedure (1.29 (0.82, 4.60); 1.50 ± 1.03 pg/mg; Fig. 2).

**Figure 3:**
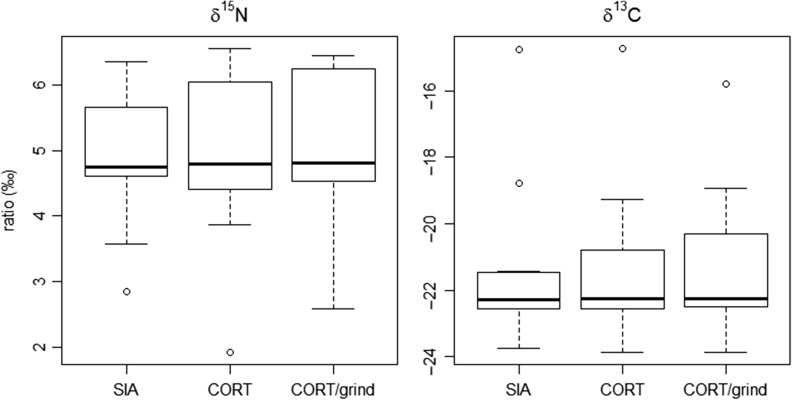
Box plot of median stable nitrogen *δ*^15^N and carbon *δ*^13^C values in hair samples of 12 brown bear individuals across different washing protocols used in preparatory stage. The *δ*^15^N values in aliquots rinsed with 2:1 chloroform:methanol mixture (SIA), were not significantly different from aliquots washed in methanol (CORT), and from the aliquots washed with methanol and ground into powder (CORT/grind), by the Wilcoxon test (*V* = 43, *P* = 0.791 and *V* = 42, *P* = 0.850, respectively). No significant difference was found in *δ*^15^N values between CORT and CORT/grind procedures (*V* = 30, *P* = 0.505). The *δ*^13^C values were not significantly different between CORT and SIA procedures (*V* = 45.5, *P* = 0.638), CORT and CORT/grind procedures (*V* = 29, *P* = 0.756) and CORT/grind and SIA procedures (*V* = 39, *P* = 0.999).

We found no significant differences in *δ*^15^N values between CORT and SIA procedures (*V* = 43, *P* = 0.791; CORT: 4.8‰ (1.9‰, 6.6‰), 4.9 ± 1.3‰; SIA: 4.7‰ (2.9‰, 6.4‰), 4.9 ± 1.0‰), CORT and CORT/grind procedures (*V* = 30, *P* = 0.505; CORT/grind: 4.8‰ (2.6‰, 6.4‰), 5.0 ± 1.2‰), or CORT/grind and SIA procedures (*V* = 42, *P* = 0.850; Fig. 3). Similarly, we found also no significant differences in *δ*^13^C values between CORT and SIA procedures (*V* = 45.5, *P* = 0.638; CORT: −22.3‰ (−23.9‰, −14.7‰), −21.4 ± 2.4‰; SIA: −22.3‰ (−23.8‰, −14.8‰), −21.5 ± 2.5‰), CORT and CORT/grind procedures (*V* = 29, *P* = 0.756; CORT/grind: −22.3‰ (−23.9‰, −15.8‰), −21.3 ± 2.2‰) and CORT/grind and SIA procedures (*V* = 39, *P* = 0.999; Fig. 3).

## Discussion and conclusions

We failed to reject our null hypotheses and found no differences in cortisol concentrations or in stable isotope values for brown bear hair washed with methanol, used in sample preparation for cortisol measurement, compared with the 2:1 chloroform:methanol rinse used for stable isotope preparations. This suggests that the chloroform:methanol rinse was adequate at cleaning the external surface of hair shafts without extracting endogenous cortisol. An important caution, however, is that hair samples used in this study were visibly clean, i.e. no obvious blood or dirt was observed on the hair shafts. It is possible that a single rinse with chloroform:methanol may not have been adequate to remove external contamination had we used hair that was significantly contaminated with blood, sebum, or dirt. There were also no differences found in stable isotope values for ground hair vs. intact hair, indicating that the standard full protocol for hair cortisol sample preparation was sufficient for obtaining reliable stable isotope ratio data.

As shown, initial preparatory procedures used for either hair cortisol or stable isotope determinations provide reliable and reproducible measurements. Furthermore, while the methods share a common basis (washing and/or grinding), the overall time in the lab would be reduced with samples prepared and processed in one laboratory and aliquots shared for the final stage of the other analysis. Shorter overall processing and thus laboratory time decrease the labor input, which also lowers personnel costs. With respect to analytical costs, we found a wide range of prices in North America and Europe (price offers available online as for mid-August 2016, converted to US$). The cost of SIA per unprepared sample ranged from $32.50 to $78 (excluding shipping), and decreased to $8.50–20 (excluding shipping) if the sample is sent fully prepared (washed, dried, weighed, cut or ground or homogenized, and packed in tin capsules). For hair cortisol analysis, the costs ranged from $35 to $55 per sample. The analytical costs can therefore decrease by about 50% when samples are sent fully prepared (washed and powdered) for final analytical procedures.

Though inter-laboratory calibrations are routine, the exercise to unify preparatory methods presented in this study is worth considering, as it could also lower the ecological footprint of the projects. The additional value comes in using archived or stored materials prepared by one protocol for the other, and pursuing hypotheses related to both assays within same study. Combining cortisol and stable isotope measurements can advance questions relevant to conservation physiology (e.g. relations of stress and nutritional status; Kempster *et al*., 2007; Deschner *et al*., 2012), and identify mechanisms underlying the patterns and trends observed in wildlife populations (e.g. Albano, 2012).

In conclusion, the findings of this study suggest that the common washing procedures and preprocessing steps used for the analyses of cortisol and stable *δ*^13^C and *δ*^15^N in animal hair do not lead to significant discrepancies in the final results. In other words, one procedure, using either a 2:1 chloroform:methanol mixture or methanol alone, can be used to wash hair in advance of determining cortisol, *δ*^13^C, and *δ*^15^N values. Further, stable isotope values can be measured using either intact hair shafts or ground hair, the latter being required for cortisol analysis. This greatly reduces the costs and labor of sample preparation, and expands the set of samples available for forensic, physiological and ecological studies.

## Author contributions

Conceived and designed the experiment: A.S., K.A.H., D.M.J., N.S., A.Z. Performed the experiments: A.S., L.K., K.A.H., C.G. Analyzed the data: A.S., M.C., A.Z. Contributed reagents/materials/analysis tools: D.M.J., K.A.H., M.C. Wrote the paper: A.S., M.C., D.M.J., A.Z., K.A.H., N.S., L.K., C.G.

## Supplementary Material

Supplementary Data

## References

[cox021C1] Accorsi PA, Carloni E, Valsecchi P, Viggiani R, Gamberoni M, Taminini C, Seren E (2008) Cortisol determination in hair and faeces from domestic cats and dogs. Gen Compar Endocrinol 155: 398–402. 10.1016/j.ygcen.2007.07.002 17727851

[cox021C2] Albano N (2012) Conservation physiology tools: their role in assessing habitat quality in birds. Ardeola 59(2): 197–216.

[cox021C3] Arnemo JM, Evans A, Fahlman A (2012) Biomedical Protocols for Free-ranging Brown Bears, Wolves, Wolverines and Lynx. Directorate for Nature Management, Trondheim, Norway.

[cox021C4] Barger CP, Kitaysky AS (2011) Isotopic segregation between sympatric seabird species increases with nutritional stress. Biol Lett 8(3): 442–445. 22171022 10.1098/rsbl.2011.1020 PMC3367740

[cox021C5] Bechshøft TØ, Rigét FF, Sonne C, Letcher RJ, Muir DCG, Novak MA, Henchey E, Meyer JS, Eulaers I, Jaspers VLB, et al (2012) Measuring environmental stress in East Greenland polar bears, 1892–1927 and 1988–2009: what does hair cortisol tell us. Environ Int 45: 15–21. 22572112 10.1016/j.envint.2012.04.005 PMC3366040

[cox021C6] Boecklen WJ, Yarnes CT, Cook BA, James AC (2011) On the use of stable isotopes in trophic ecology. Annu Rev Ecol Evol Syst 42: 411–440.

[cox021C7] Bontempo L, Ceppa F, Ziller L, Pedrini P, Hobson KA, Wassenaar LI, Camin F (2014) Comparison of methods for stable isotope ratio (*δ*^13^C, *δ*^15^N, *δ*^2^H, *δ*^18^O) measurements of feathers. Methods Ecol Evol 5: 362–371.

[cox021C8] Bortolotti GR, Marchant TA, Blas J, Cabezas S (2009) Tracking stress: localisation, deposition and stability of corticosterone in feathers. J Exp Biol 212: 1477–1482. 19411541 10.1242/jeb.022152

[cox021C9] Bowen GJ, Wassenaar LI, Hobson KA (2005) Global application of stable hydrogen and oxygen isotopes to wildlife forensics. Oecologia 143: 337–348. 15726429 10.1007/s00442-004-1813-y

[cox021C10] Bryan HM, Dairmont CT, Paquet PC, Wynne-Edwards KE, Smits JEG (2013) Stress and reproductive hormones in grizzly bears reflect nutritional benefits and social consequences of a salmon foraging niche. PLoS One 8(11): e80537. 24312230 10.1371/journal.pone.0080537 PMC3842319

[cox021C11] Cattet M, Macbeth BJ, Janz DM, Zedrosser A, Swenson EJ, Dumond M, Stenhouse GB (2014) Quanifying long-term stress in brown bears with the hair cortisol concentration: a biomarker that may be confounded by rapid changes in response to capture and handling. Conserv Physiol 2: doi:10.1093/conphys/cou026. 10.1093/conphys/cou026 PMC4732478 27293647

[cox021C12] Comin A, Prandi A, Peric T, Corazzin M, Dovier S, Bovolenta S (2011) Hair cortisol levels in dairy cows from winter housing to summer highland grazing. Livest Sci 138: 69–73.

[cox021C13] Crawford K, McDonals RA, Bearhop S (2008) Applications of stable isotope techniques to the ecology of mammals. Mammal Rev 38(1): 87–107.

[cox021C14] Darimont CT, Paquet PC, Reimchen TE (2007) Stable isotopic niche predicts fitness of prey in a wolf-deer system. Biol J Linnean Soc 90: 125–137.

[cox021C15] Davenport MD, Tiefenbacher S, Lutz CK, Novak MA, Meyer JS (2006) Analysis of endogenous cortisol concentrations in the hair of rhesus macaques. Gen Compar Endocrinol 147: 255–261. 10.1016/j.ygcen.2006.01.005 16483573

[cox021C16] Deschner T, Fuller BT, Oelze VM, Boesch C, Hublin JJ, Mundry R, Richards MP, Ortmann S, Hohmann G (2012) Identification of energy consumption and nutritional stress by isotopic and elemental analysis of urine in bonobos (*Pan paniscus*). Rapid Commun Mass Spectrom 26: 69–77. 22215580 10.1002/rcm.5312

[cox021C17] Eser HP, Pötsch L, Skopp G, Moeller MR (1997) Influence of sample preparation on analytical results: drug analysis [GC/MS] on hair snippets versus hair powder using various extraction methods. Forensic Sci Int 84: 271–279. 9042733 10.1016/s0379-0738(96)02071-3

[cox021C18] Fairhurst GD, Bond AL, Hobson KA, Ronconi RA (2014) Feather-based measures of stable isotopes and corticosterone reveal a relationship between trophic position and physiology in a pelagic seabird over 153-year period. IBIS 2014: 12232.

[cox021C19] Font L, Nowell GM, Graham Pearson D, Ottley CJ, Willis SG (2007). Sr isotope analysis of bird feathers by TIMS: a tool to trace bird migration paths and breeding sites. J Anal Atom Spectrom 22: 513–522.

[cox021C20] Hobson KA, McLellan BN, Woods JG (2000) Using stable carbon (*δ*^13^C) and nitrogen (*δ*^15^N) isotopes to infer trophic relationships among black and grizzly bears in the upper Columbia River basin, British Columbia. Can J Zool 78: 1332–1339.

[cox021C21] Hobson KA, Wassenaar LI, eds (2008) Tracking animal migration using stable isotopes Handbook of Terrestrial Ecology Series, Academic Press/Elsevier, Amsterdam, p 188.

[cox021C22] Jenni-Eiermann S, Helfenstein F, Vallat A, Glauser G, Jenni L (2015) Corticosterone: effects on feather quality and deposition into feathers. Methods Ecol Evol 6: 237–246.

[cox021C23] Kempster B, Zanette L, Longstaffe FJ, MacDougal-Shackleton SA, Wingfield JC, Clinchy M (2007) Do stable isotopes reflect nutritional stress? Results from a laboratory experiment on song sparrows. Oecologia 151(3): 365–371. 17102993 10.1007/s00442-006-0597-7

[cox021C24] Koren L, Mokady O, Karaskovi T, Klein J, Koren G, Geffen E (2002) A novel method using hair for determining hormonal levels in wildlife. Anim Behav 63: 403–406.

[cox021C25] Lafferty DJR, Laudenslager ML, Mowat G, Heard D, Belant JL (2015) Sex, diet, and the social environment: factors influencing hair cortisol concentration in free-ranging black bears (*Ursus americanus*). PLoS ONE 10(11): e0141489. 26529405 10.1371/journal.pone.0141489 PMC4631324

[cox021C26] Macbeth BJ, Cattet MRL, Stenhouse GB, Gibeau ML, Janz DM (2010) Hair cortisol concentration as a noninvasive measure of long-term stress in free-ranging grizzly bears (*Ursus arctos*): considerations with implications for other wildlife. Can J Zool 88(10): 935–949.

[cox021C27] Narum SR (2006) Beyond Bonferroni: less conservative analyses for conservation genetics. Conserv Genet 7: 783–787.

[cox021C28] Newsome SD, Martínez del Rio C, Bearhop S, Phillips DL (2007) A niche for isotopic ecology. Frontiers Ecol Environ 5: 429–436.

[cox021C29] Newsome SD, Ralls K, Van Horn Job C, Fogel ML, Cypher BL (2010) Stable isotopes evaluate exploitation of anthropogenic foods by the endangered San Joaquin kit fox (*Vulpes macrotis mutica*). J Mammal 91(6): 1313–1321.

[cox021C30] Newsome SD, Tinker MT, Monson DH, Oftedal O, Ralls K, Fogel ML, Estes JA (2009). Using stable isotopes to investigate individual diet specialization in California sea otters (*Enhydra lutris nereis*). Ecology 90(4): 961–974. 19449691 10.1890/07-1812.1

[cox021C31] R Core Team (2016) R: A language and Environment for Statistical Computing. R Foundation for Statistical Computing, Vienna, Austria URL https://www.R-project.org/.

[cox021C32] Riofrío-Lazo M, Páez-Rosas D (2015) Feeding habits of introduced black rats, *Rattus rattus*, in nesting colonies of Galapagos petrel on San Cristóbal Island, Galapagos. PLoS One 10(5): e0127901. 25984724 10.1371/journal.pone.0127901 PMC4436216

[cox021C33] Russel E, Koren G, Rieder M, Van Uum SH (2014) The detection of cortisol in human sweat: implications for measurement of cortisol in hair. Ther Drug Monit 36(1): 30–34. 24216536 10.1097/FTD.0b013e31829daa0a

[cox021C34] Stalder T, Kirschbaum C (2012) Analysis of cortisol in hair—state of the art and future directions. Brain Behav Immun 26(7): 1019–1029. 22366690 10.1016/j.bbi.2012.02.002

[cox021C35] Wassenaar LI (2008) An introduction to light stable isotopes for use in terrestrial animal migration studies In: Hobson KA, Wassenaar LI, eds, Tracking Animal Migration with Stable Isotopes, Ed 1 Vol 2 Terrestrial Ecology, Academic Press, London, pp 21–41.

[cox021C36] Webb E, Thomson S, Nelson A, White C, Koren G, Rieder M (2010) Assessing individual systemic stress through cortisol analysis of archaeological hair. J Archaeol Sci 37: 807–812.

[cox021C37] Wikelski M, Cooke SJ (2006) Conservation physiology. Trends Ecol Evol 21(1): 38–46. 16701468 10.1016/j.tree.2005.10.018

