# Supplementary material for: Compatibility of preparatory procedures for the analysis of cortisol concentrations and stable isotope (δ13C, δ15N) ratios: a test on brown bear hair
Source: Conserv Physiol. 2017 Mar 24;5(1):cox021. doi: 10.1093/conphys/cox021 (PMC5569930; doi:10.1093/conphys/cox021)

**Supplementary material** to:

**Compatibility of preparatory procedures for the analysis of cortisol concentrations and stable isotope (*δ*^13^C, *δ*^15^N) ratios: a test on brown bear hair**

**Agnieszka Sergiel, Keith A. Hobson, David M. Janz, Marc Cattet, Nuria Selva, Luciene Kapronczai, Chantel Gryba, and Andreas Zedrosser**

**Comparison of the fraction of C (%) between washing treatments**

We found no significant differences in the fractions of C between CORT and SIA procedures (*V* = 59, *p*-value = 0.129), CORT and CORT/grind procedures (*V* = 17, *p*-value = 0.092) or CORT/grind and SIA procedures (*V* = 30, *p*-value = 0.519).

**Comparison of the fraction of N (%) between washing treatments**

We found no significant differences in the fractions of N between CORT and SIA procedures (*V* = 41, *p*-value = 0.505), CORT and CORT/grind procedures (*V* = 15, *p*-value = 0.120) or CORT/grind and SIA procedures (*V* = 18, *p*-value = 0.108).

**Comparison of the C:N ratio between washing treatments**

We found no significant differences in the C:N ratio between CORT and SIA procedures (*V* = *32*, *p*-value = 0.964) or CORT and CORT/grind procedures (*V* = 51, *p*-value = 0.119). We found that the C:N ratio was significantly higher with the CORT/grind procedure (median: 3.005, range: 2.97 – 3.09) in comparison to the SIA procedures (median: 2.995, range: 2.91 – 3.06; *V* = 64.5, *p*-value = 0.0493), however, this significant difference disappeared after the removal of an outlier (*V* = 14, *p*-value = 0.125).

**Supplementary table 1:** Comparison of *δ* ^13^C, *δ* ^15^N (‰) and cortisol concentration (pg/mg) values measured in hair samples of twelve brown bear individuals. In stable isotopes analysis aliquots were chloroform:methanol rinsed (SIA), methanol washed (CORT) or methanol washed and ground (CORT/grind), and analysed following stable isotopes analysis protocol. In cortisol analysis aliquots were chloroform:methanol rinsed (SIA) or methanol washed (CORT) and then processed further and analyzed following cortisol concentration measurement protocol.

| Sample ID | *δ* ^13^C (‰) | | |  | *δ* ^15^N (‰) | | |  | Cortisol (pg/mg) | |
| --- | --- | --- | --- | --- | --- | --- | --- | --- | --- | --- |
|  | SIA | CORT | CORT/grind |  | SIA | CORT | CORT/grind |  | SIA | CORT |
| 1 | -22.5 | -22.4 | -22.5 |  | 4.7 | 5.0 | 4.9 |  | 1.30 | 1.46 |
| 2 | -22.2 | -22.3 | -22.3 |  | 4.6 | 4.6 | 4.5 |  | 0.97 | 1.34 |
| 3 | -22.4 | -22.2 | -22.3 |  | 5.5 | 4.9 | 5.0 |  | 1.39 | 1.23 |
| 4 | -22.6 | -22.6 | -22.6 |  | 5.8 | 6.6 | 6.4 |  | 1.22 | 0.94 |
| 5 | -22.6 | -22.5 | -22.5 |  | 5.3 | 5.9 | 6.3 |  | 1.04 | 1.37 |
| 6 | -22.3 | -22.1 | -22.2 |  | 3.6 | 3.9 | 3.8 |  | 1.66 | 1.96 |
| 7 | -23.5 | -22.6 | -22.2 |  | 2.9 | 1.9 | 2.6 |  | 1.63 | 1.42 |
| 8 | -21.5 | -21.6 | -21.5 |  | 4.6 | 4.3 | 4.6 |  | 1.16 | 1.00 |
| 9 | -18.8 | -19.3 | -18.9 |  | 4.8 | 4.7 | 4.7 |  | 0.66 | 0.94 |
| 10 | -23.8 | -23.9 | -23.9 |  | 4.7 | 4.5 | 4.5 |  | 6.69 | 4.60 |
| 11 | -14.8 | -14.7 | -15.8 |  | 6.4 | 6.5 | 6.2 |  | 0.69 | 0.82 |
| 12 | -21.4 | -20.0 | -19.1 |  | 5.9 | 6.2 | 6.3 |  | 1.06 | 0.92 |

**Supplementary table 2:** Descriptive statistics for *δ* ^13^C, *δ* ^15^N and cortisol values measured in hair samples of twelve brown bear individuals, subsampled and processed using different protocols. In stable isotopes analysis aliquots were chloroform:methanol rinsed (SIA), methanol washed (CORT) or methanol washed and ground (CORT/grind), and analysed following stable isotopes analysis protocol. In cortisol analysis aliquots were chloroform:methanol rinsed (SIA) or methanol washed (CORT) and then processed further and analyzed following cortisol concentration measurement protocol.

|  |  | *δ* ^13^C (‰) | | |  | *δ* ^15^N (‰) | | |  | Cortisol (pg/mg) | |
| --- | --- | --- | --- | --- | --- | --- | --- | --- | --- | --- | --- |
|  |  | SIA | CORT | CORT/grind |  | SIA | CORT | CORT/grind |  | SIA | CORT |
| Min |  | -23.8 | -23.9 | -23.9 |  | 2.8 | 1.9 | 2.6 |  | 0.66 | 0.82 |
| Max | | -14.8 | -14.7 | -15.8 |  | 6.4 | 6.6 | 6.4 |  | 6.69 | 4.60 |
| Mean | | -21.5 | -21.4 | -21.3 |  | 4.9 | 4.9 | 5.0 |  | 1.62 | 1.50 |
| Median | | -22.3 | -22.3 | -22.3 |  | 4.7 | 4.8 | 4.8 |  | 1.19 | 1.28 |
| SD | | 2.5 | 2.4 | 2.2 |  | 1.0 | 1.3 | 1.2 |  | 1.63 | 1.03 |

**Supplementary figure 1:** Concordance plot demonstrating the correlation between hair cortisol concentration values (pg/mg) obtained after cleaning procedure used in stable isotope analysis (SIA) and cortisol concentration measurement (CORT).


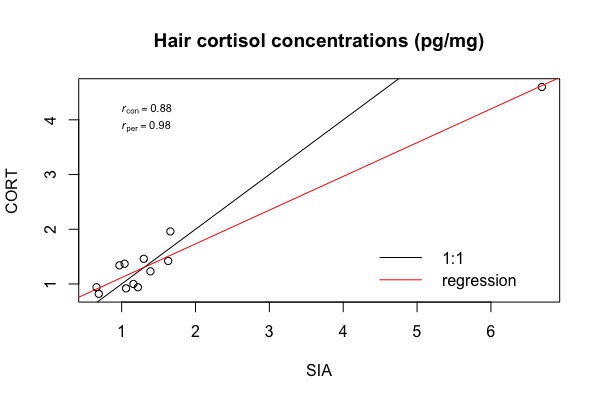


**Supplementary figure 2:** Concordance plots demonstrating the correlation between stable carbon (top) and nitrogen ratios (bottom) obtained after cleaning procedures used in stable isotope analysis (SIA) and cortisol concentration measurement (CORT and CORT/grind). Red: regression line; black: line of perfect concordance.


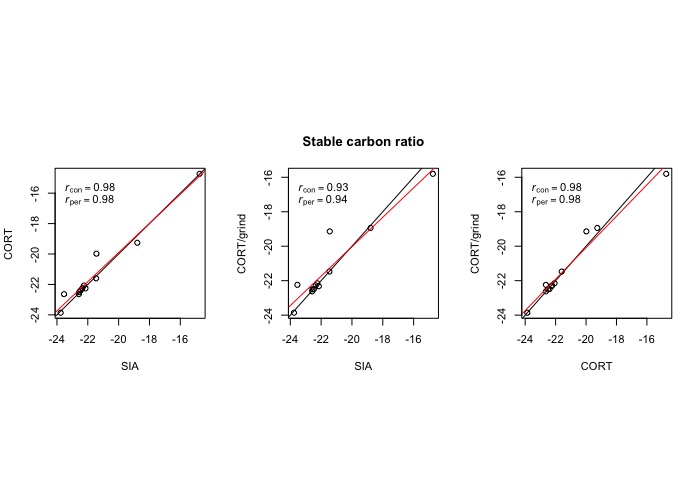


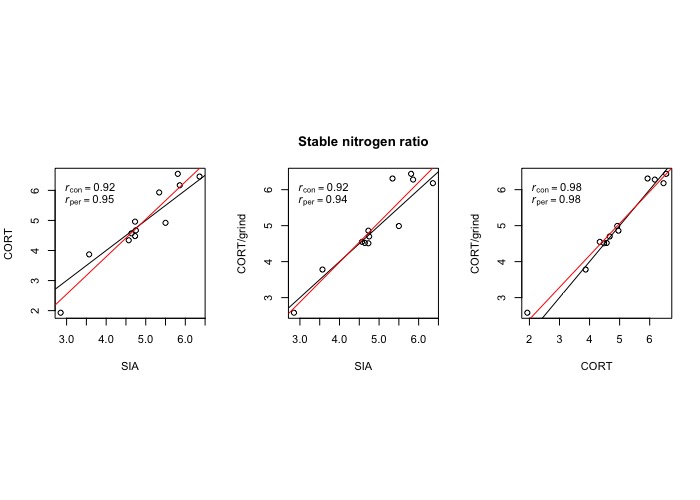

Supplement: Supplementary Data [file Supplementarymaterial_SergieletalCompatibilityofpreparatoryprocedures.docx]
